# Design and Analysis of a Dual-Band Implantable Receiving Antenna for Wireless Power Transfer and Data Communication at 1.32 GHz and 2.58 GHz

**DOI:** 10.3390/s25247507

**Published:** 2025-12-10

**Authors:** Ashfaq Ahmad, Sun-Woong Kim, Dong-You Choi

**Affiliations:** 1 Department of Information and Communication Engineering, Chosun University, Gwangju 61452, Republic of Korea; 2 National Center of Excellence in Software, Chosun University, Gwangju 61452, Republic of Korea

**Keywords:** implantable antenna, wireless power transfer, biotelemetry systems

## Abstract

This paper presents the design and performance evaluation of a compact dual-band implantable antenna (Rx) operating at 1.32 GHz and 2.58 GHz for biomedical applications. The proposed antenna is designed to receive power and data from an external transmitting (Tx) antenna operating at 1.32 GHz. The measured impedance bandwidths of the Rx antenna are 190 MHz (1.23–1.42 GHz) and 230 MHz (2.47–2.70 GHz), covering both the power transfer and data communication bands. The wireless power transfer efficiency, represented by the transmission coefficient ($S_{21}$), is observed to be −40 dB at a spacing of 40 mm, where the Rx is located in the far-field region of the Tx. Specific Absorption Rate (SAR) analysis is performed to ensure electromagnetic safety compliance, and the results are within the acceptable exposure limits. The proposed antenna achieves a realized gain of −25 dB at 1.32 GHz and −25.8 dB at 2.58 GHz, demonstrating suitable performance for low-power implantable medical device communication and power transfer systems. The proposed design offers a promising solution for reliable biotelemetry and wireless power transfer in implantable biomedical systems.

## 1. Introduction

The rapid evolution of the Internet of Medical Things (IoMT) has driven an increasing demand for miniaturized, power-efficient, and reliable implantable medical devices (IMDs) capable of performing continuous monitoring and therapeutic functions without physical connections or frequent surgical interventions [1,2,3]. Among the various enabling technologies, wireless power transfer (WPT) has emerged as a critical solution to overcome the limitations of traditional battery-based systems by providing sustainable and non-invasive energy delivery to deeply implanted biomedical sensors and stimulators [4,5]. The integration of WPT with biotelemetry facilitates seamless power and data exchange, supporting the realization of next-generation battery-free healthcare systems. However, the design of efficient implantable antennas for WPT applications remains challenging due to the stringent constraints imposed by the human body environment. Biological tissues exhibit high dielectric constants and conductive losses, leading to significant attenuation and impedance mismatching between the implanted and external antennas [6,7]. Furthermore, miniaturization requirements necessitate compact antenna geometries that often compromise bandwidth, gain, and radiation efficiency [8,9]. Achieving stable operation across multiple frequency bands while maintaining electromagnetic safety within Specific Absorption Rate (SAR) limits adds further complexity to the design process [10,11,12]. To address these challenges, researchers have explored various optimization strategies for enhancing coupling and radiation efficiency in implantable systems. The introduction of dielectric matching media (MM) has demonstrated substantial improvements in near-field coupling and power transfer efficiency by reducing impedance discontinuities between air and biological tissues [13,14]. Similarly, metamaterial-based and machine learning-assisted WPT designs have been reported to further enhance energy focusing and material selection for improved in-body propagation [15,16,17]. Although these methods significantly advance the field, many existing designs rely on intricate fabrication processes or operate within limited bandwidths, constraining their applicability to multi-functional medical platforms. In this context, dual-band implantable antennas offer a promising alternative, enabling simultaneous energy harvesting and data telemetry using distinct frequency bands [18]. The lower band is typically optimized for power transfer, while the higher band facilitates high-rate communication with external medical infrastructure. Such dual-mode architectures ensure the efficient use of available spectrum and improve the robustness of biotelemetric links, particularly in dynamic tissue environments. Motivated by these considerations, this paper presents the design and analysis of a compact dual-band implantable receiving (Rx) antenna operating at 1.32 GHz and 2.58 GHz, optimized for wireless power transfer and data communication in biomedical applications. The proposed antenna employs a double-slot configuration with an additional fine-tuning slot and a via-connected ground structure to achieve effective miniaturization and impedance matching in lossy tissue environments. The lower band (1.32 GHz) supports efficient wireless power transfer from an external transmitter, whereas the upper band (2.58 GHz) enables high-frequency biotelemetry. Comprehensive full-wave simulations and parametric analyses are conducted to investigate the effects of feed position, via geometry, and tissue loading on the antenna performance. Furthermore, SAR evaluations confirm compliance with international safety standards, validating the antenna’s suitability for implantable operation. The remainder of this paper is organized as follows. Section 2 discusses the detailed design and analysis of the proposed system, including the overall design overview and the development of both the Tx and Rx antennas along with their parametric analyses. Section 3 presents the simulation results and discussion, highlighting the antenna performance in various configurations. Finally, Section 4 concludes the paper with a summary of the key findings and future research directions.

## 2. Design and Analysis

### 2.1. Design Overview

**Figure 1:**
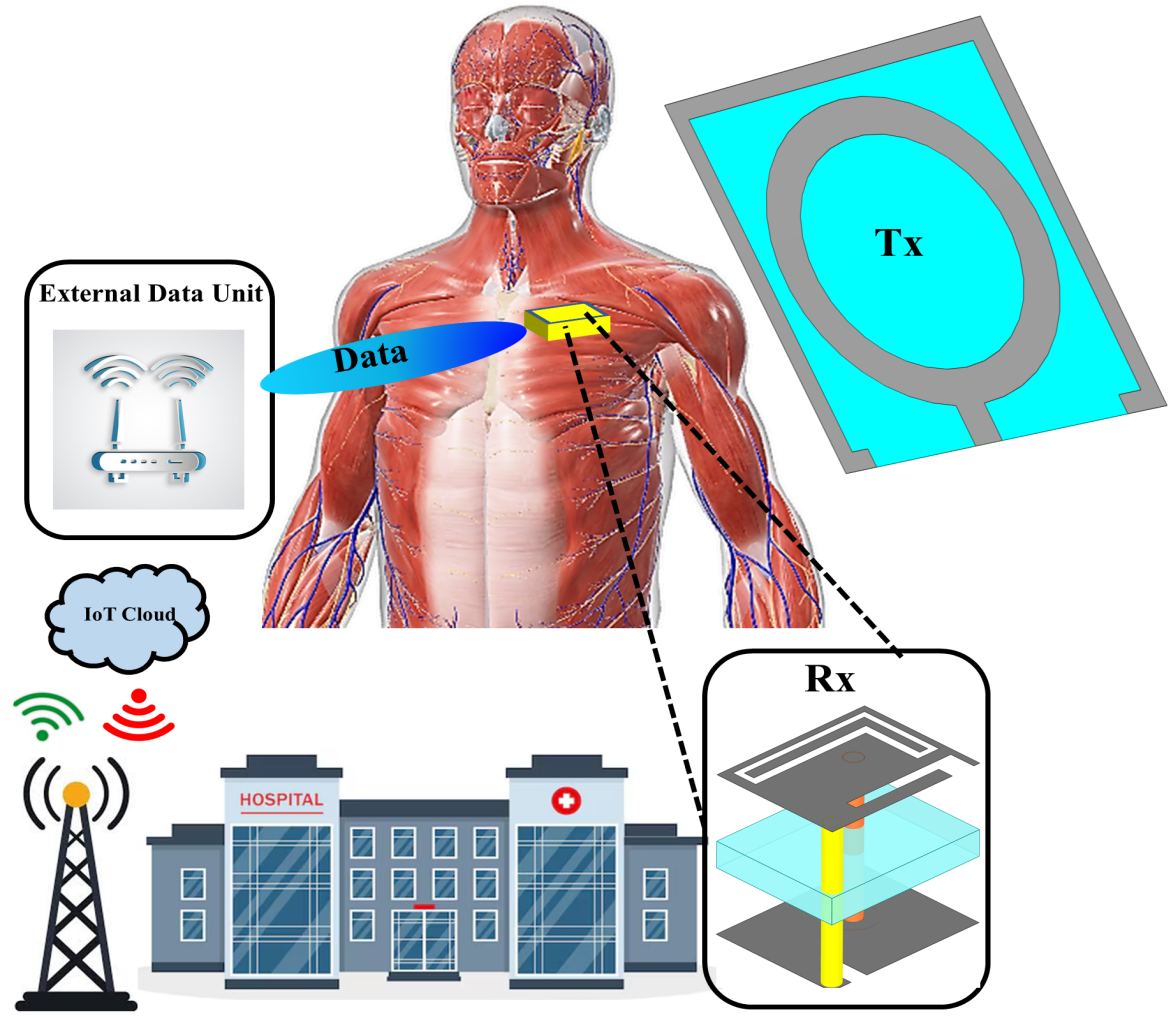
Schematic illustration of the proposed wirelessly powered implantable system, showing the external transmitting (Tx) antenna and the implanted receiving (Rx) antenna integrated within the multilayer tissue model.

The proposed system consists of an external transmitting (Tx) antenna and an implantable receiving (Rx) antenna, as illustrated in Figure 1. The Tx antenna delivers continuous wireless power at 1.32 GHz to the implanted Rx antenna, while bidirectional data transfer is achieved through the higher 2.58 GHz band. Both antennas are designed and optimized to achieve proper impedance matching, stable radiation characteristics, and compliance with electromagnetic safety limits defined by SAR (Specific Absorption Rate) standards within biological tissue. The overall design procedure involves parametric optimization of antenna geometry, careful substrate selection to ensure biocompatibility, and full-wave electromagnetic simulation using a multilayer human tissue model comprising skin, fat, and muscle layers. These simulations aid in understanding the field distribution, power coupling efficiency, and the impact of tissue loading on antenna performance.

### 2.2. Design and Analysis of Rx Antenna

**Figure 2:**
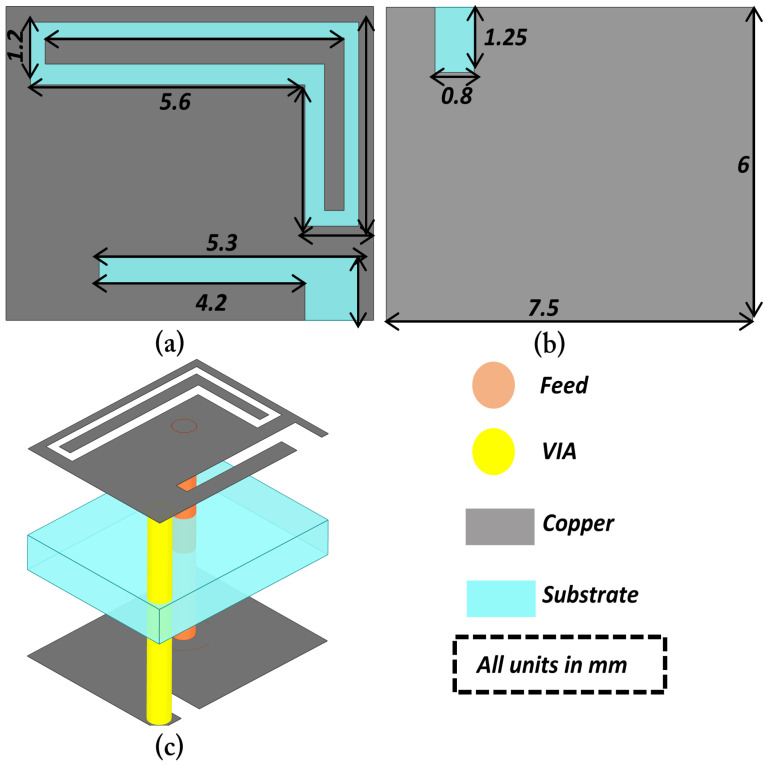
Eometry of the proposed dual-band implantable receiving (Rx) antenna designed on the Rogers RT/Duroid 6010 substrate: (**a**) Front view; (**b**) Back view; (**c**) Isometric view.

**Figure 3:**
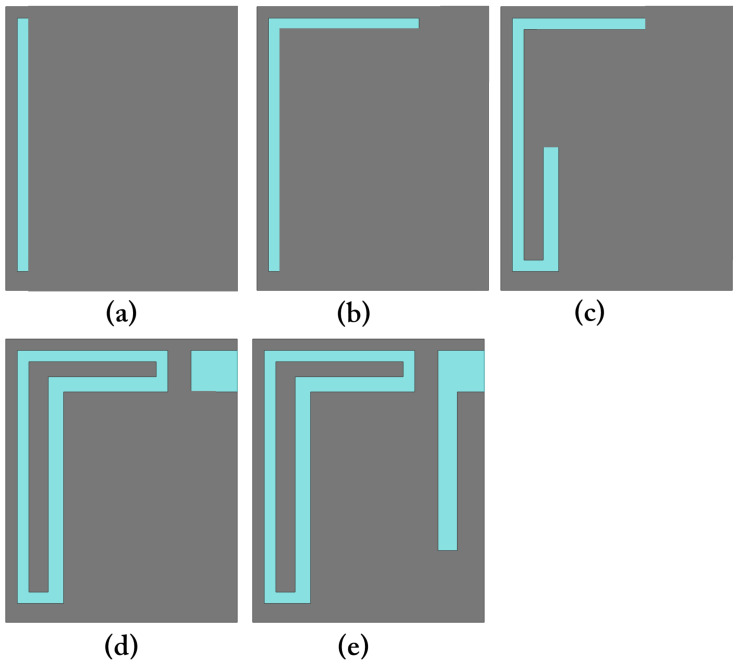
Step-by-step geometrical evolution of the proposed Rx antenna: (**a**) Initial structure; (**b**) First modification; (**c**) Second modification; (**d**) Slot introduction; (**e**) Final optimized dual-slot design.

**Figure 4:**
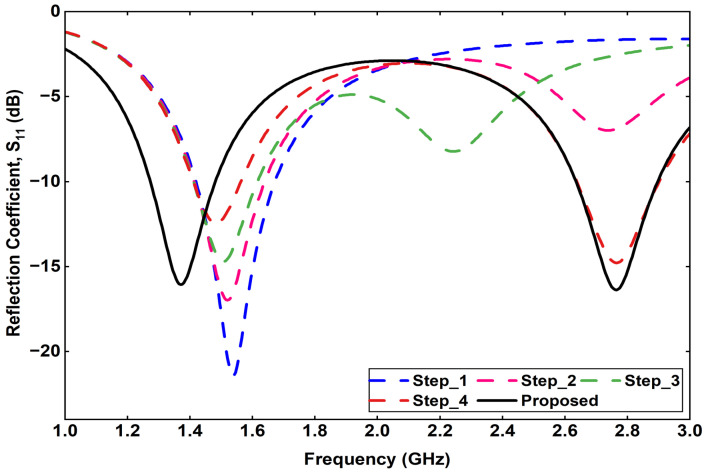
Step-by-Step Simulated reflection coefficient.

The proposed implantable receiving (Rx) antenna is designed to achieve dual-band operation, compactness, and biocompatibility while maintaining good impedance matching in the tissue environment. To realize the dual-band characteristics, a double-slot structure is introduced on the main radiating patch, and an additional small slot is etched to further fine-tune the upper resonant frequency and improve bandwidth. A via is employed to connect the patch to the ground plane, effectively contributing to antenna miniaturization by introducing inductive loading. The overall geometry of the proposed Rx antenna, including the front view, back view, and isometric perspective, is illustrated in Figure 2. The antenna design is developed through a systematic five-step evolution process, denoted as Steps (a)–(e), as depicted in Figure 3. Each step represents a key modification in the geometry aimed at improving impedance matching and achieving the desired dual-band performance. The corresponding reflection coefficient ($S_{11}$) responses for each design stage are shown in Figure 4, demonstrating the gradual evolution toward optimized resonance at 1.32 GHz and 2.58 GHz.

**Figure 5:**
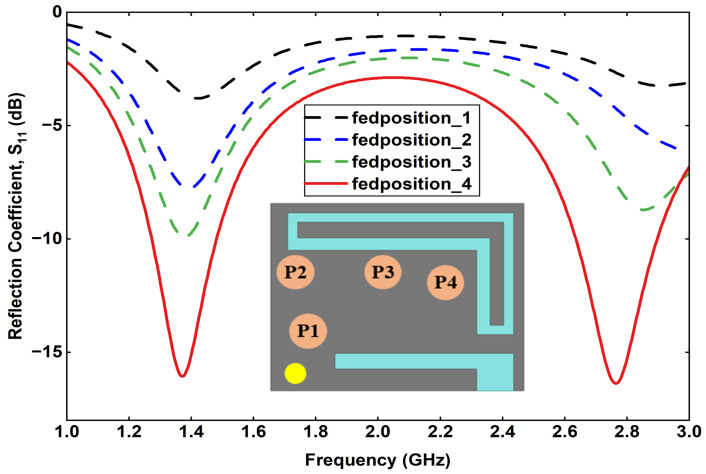
Effect of feed position variation on the reflection coefficient.

**Figure 6:**
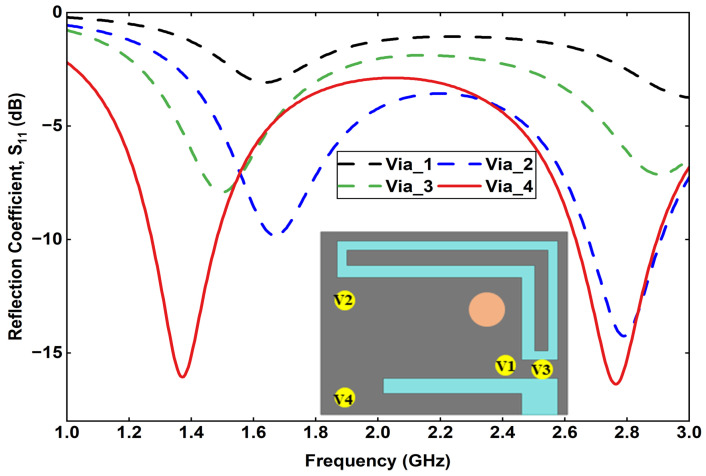
Effect of via position variation on the reflection coefficient.

**Figure 7:**
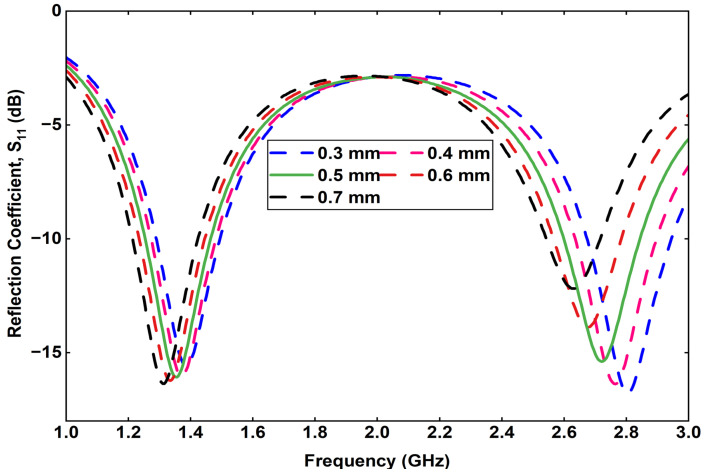
Variation in the reflection coefficient by changing the via size.

Furthermore, a detailed parametric analysis is conducted on the three most critical parameters affecting the antenna performance: feed position, via position, and via diameter. The results of these analyses are summarized. Figure 5 presents the effect of varying the feed position at four different points on the radiating patch. The corresponding reflection coefficient curves indicate that a slight shift in the feed point significantly influences the impedance matching and lower-band resonance. Similarly, Figure 6 illustrates the impact of varying the via position, where each configuration yields a distinct $S_{11}$ response, affecting both the resonant frequencies and bandwidth. Finally, Figure 7 shows the effect of changing the via diameter from 0.3 mm to 0.7 mm. The results demonstrate that increasing the via width alters the inductive coupling between the patch and ground, thereby tuning the resonance frequencies and improving impedance matching.

The Rx antenna is designed on a Rogers RT/Duroid 6010 substrate, chosen for its high dielectric constant ($\varepsilon_{r}=10.2$) and low loss tangent (tanδ=0.0035), which enable antenna miniaturization while maintaining acceptable efficiency in lossy tissue environments. The substrate thickness is optimized to achieve a balance between bandwidth and gain, ensuring stable operation at both 1.32 GHz and 2.58 GHz. Overall, the introduction of slots and vias, together with systematic parametric optimization, allows the proposed Rx antenna to achieve compact size, dual-band functionality, and improved impedance performance suitable for far-field wireless power and data transfer applications.

### 2.3. Design and Analysis of Tx Antenna

**Figure 8:**
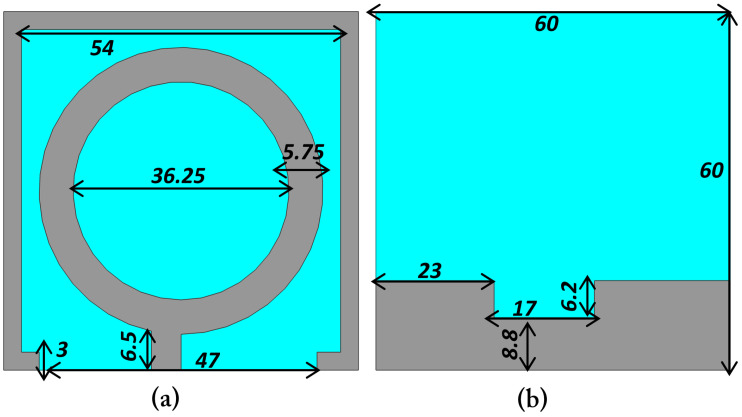
Configuration of the external transmitting (Tx) antenna showing (**a**) front view with circular ring-shaped radiator and (**b**) back view with optimized ground-plane slots and notches.

The external transmitting (Tx) antenna is designed to operate at 1.32 GHz and is primarily used for wireless power transmission to the implanted Rx antenna. The geometry of the proposed Tx antenna is shown in Figure 8, illustrating both the front and back views of the design. The antenna is designed on an *FR-4* substrate with an overall size of 60mm×60mm, a relative dielectric constant of $\varepsilon_{r}=4.4$, and a loss tangent of tanδ=0.02. The use of FR-4 material provides a cost-effective and mechanically stable platform suitable for external wearable or handheld applications.

The radiating element adopts a circular ring-shaped structure with an outer dimension of 54 mm and an inner diameter of 18.25 mm, as shown in Figure 8a. The ring width of 5.75 mm is optimized to achieve the desired resonant frequency of 1.32 GHz, while maintaining good impedance matching. The feed line is connected to the ring through a short microstrip section, and a narrow ground slot is introduced on the backside to improve radiation efficiency and impedance characteristics. The backside ground plane dimensions and notches are depicted in Figure 8b, where the cutout dimensions of 17 mm, and 6.2 mm are carefully optimized to achieve efficient coupling and stable resonance. The circular configuration of the Tx antenna was selected to provide an omnidirectional radiation pattern and uniform energy distribution toward the implant site, irrespective of the implant orientation. Since the main research focus of this work is the design and optimization of the implantable Rx antenna, the Tx antenna was kept relatively simple in geometry while ensuring sufficient gain and bandwidth for effective far-field wireless power transfer at 1.32 GHz.

## 3. Results and Discussion

**Figure 9:**
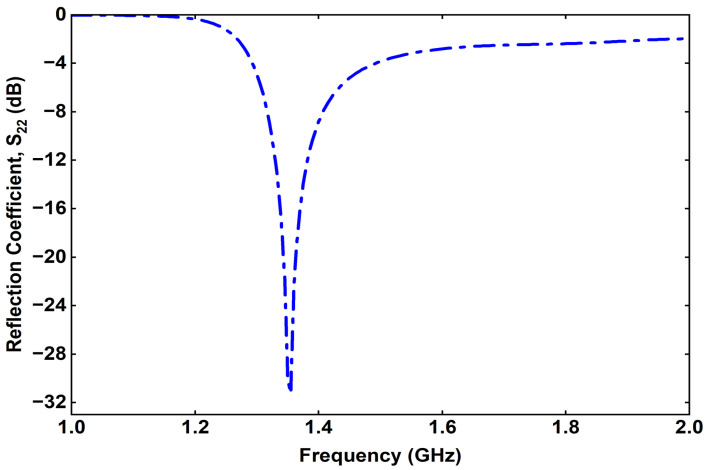
Simulated reflection coefficient of the proposed Tx.

The proposed external transmitting (Tx) antenna was designed and simulated using a full-wave electromagnetic solver to evaluate its reflection characteristics, gain, and radiation performance. The simulated S-parameter response of the Tx antenna is presented in Figure 9. It can be observed that the antenna exhibits a well-defined resonance at 1.32 GHz with a reflection coefficient ($S_{11}$) below −20 dB, indicating excellent impedance matching at the desired operating frequency. The impedance bandwidth, defined by the −10 dB criterion, adequately covers the 1.32 GHz band intended for wireless power transfer to the implantable Rx antenna.

**Figure 10:**
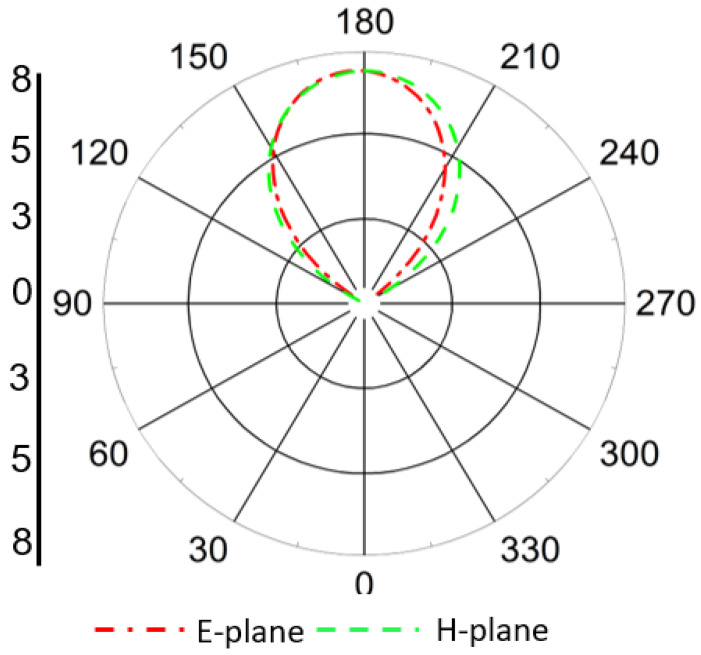
Simulated far-field radiation pattern of the proposed Tx antenna at 1.32 GHz in both E- and H-planes, showing broad, directional radiation toward the implant region.

The simulated far-field radiation pattern of the Tx antenna at 1.32 GHz is shown in Figure 10. It is evident from the figure that the proposed Tx antenna exhibits a broad, stable radiation pattern with a predominant directional lobe normal to the antenna surface, which ensures efficient power delivery toward the implanted Rx device. The antenna demonstrates good symmetry in both the E-plane and H-plane radiation characteristics, confirming its suitability for far-field energy transfer applications.

The maximum realized gain of the proposed Tx antenna is observed to be 7.38 dBi at 1.32 GHz. This relatively high gain value ensures adequate radiated power density at the implant location, thereby improving the wireless power transfer efficiency without exceeding SAR limits in surrounding tissues. The stable radiation behavior and high gain of the Tx antenna contribute significantly to the overall performance of the Tx–Rx link.

**Figure 11:**
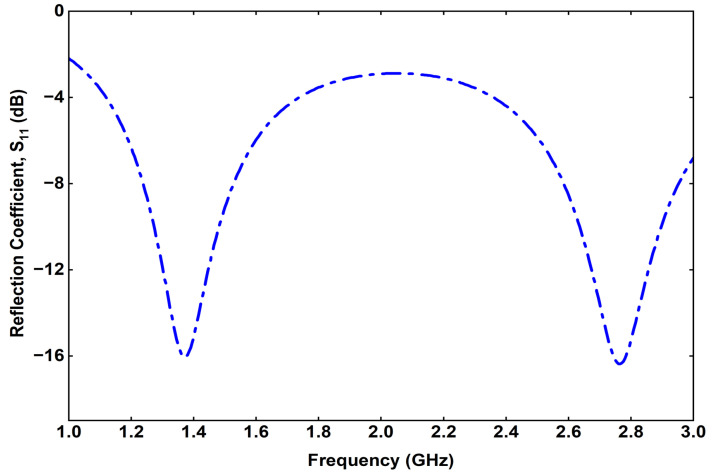
Simulated reflection coefficient of the proposed Rx.

The reflection coefficient ($S_{11}$) of the proposed implantable Rx antenna is illustrated in Figure 11. It can be observed that the antenna exhibits dual resonances at 1.32 GHz and 2.58 GHz, corresponding to the power transfer and data communication bands, respectively. The measured impedance bandwidths (for $S_{11}\leq -10\;\mathrm{dB}$) are 190 MHz in the lower band (1.23–1.42 GHz) and 230 MHz in the higher band (2.47–2.70 GHz), which sufficiently cover the desired operating frequencies. These results confirm that the designed Rx antenna successfully achieves dual-band operation with good impedance matching.

**Figure 12:**
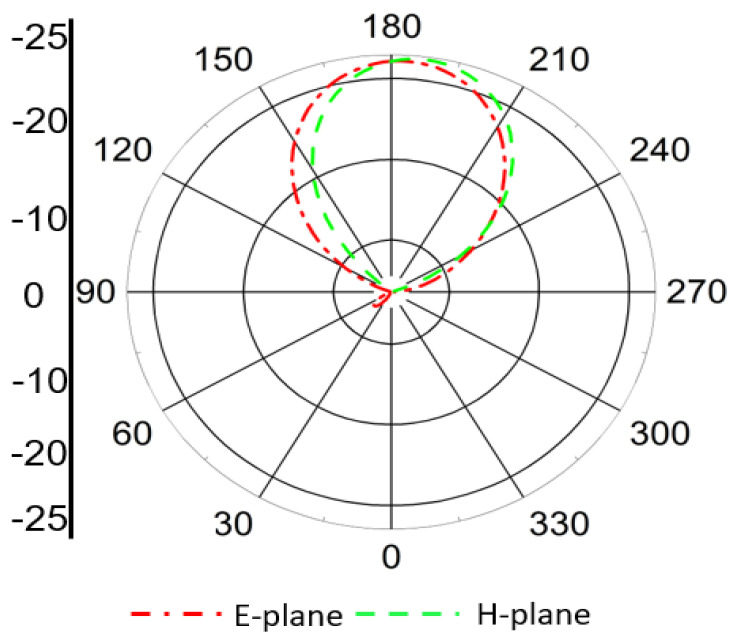
Simulated radiation pattern of the Rx antenna at 1.32 GHz showing nearly omnidirectional distribution, suitable for stable power reception under orientation variations.

**Figure 13:**
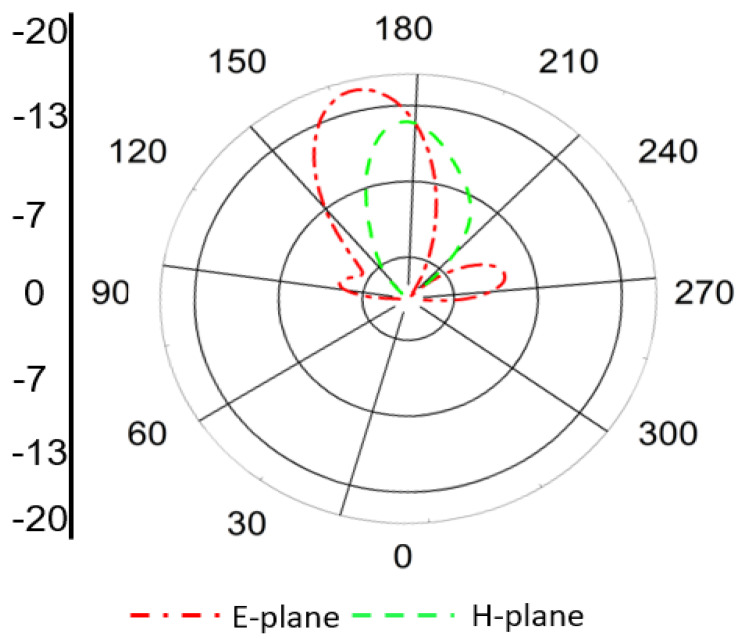
Simulated radiation pattern of the Rx antenna at 2.58 GHz showing moderately directive behavior, optimized for data telemetry performance.

The radiation patterns of the Rx antenna at both operating frequencies are presented in Figure 12 and Figure 13. At 1.32 GHz, the radiation pattern exhibits an almost omnidirectional distribution, which is beneficial for maintaining consistent power reception regardless of the implant orientation. At 2.58 GHz, the antenna demonstrates a slightly more directive radiation pattern due to the excitation of higher-order modes. This behavior enhances the antenna’s performance for data communication purposes, where higher directivity can improve link stability and transmission quality.

**Figure 14:**
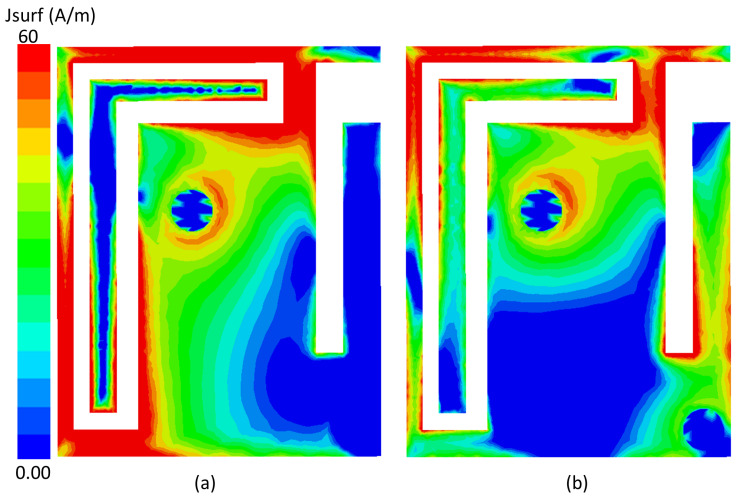
Current distribution of the proposed implantable antenna at (**a**) 1.32 GHz and (**b**) 2.58 GHz.

The current distribution of the proposed implantable antenna is shown in Figure 14. Figure 14a illustrates the current distribution at 1.32 GHz, where the maximum current is observed at the corner of the antenna, indicating a longer electrical path. In contrast, the current distribution at 2.58 GHz, shown in Figure 14b, is concentrated near the feed line, reflecting a shorter effective path at the higher frequency. These distributions confirm the dual-band behavior of the antenna and highlight the differing resonant modes at the two operating frequencies.

The realized gain of the Rx antenna is found to be −25 dB at 1.32 GHz and −22 dBi at 2.58 GHz. These gain values are consistent with expectations for miniaturized implantable antennas operating within lossy biological tissues, where high dielectric loading and absorption significantly affect radiation efficiency. Despite the reduced gain, the antenna maintains stable radiation behavior and sufficient link performance when paired with the external Tx antenna.

**Figure 15:**
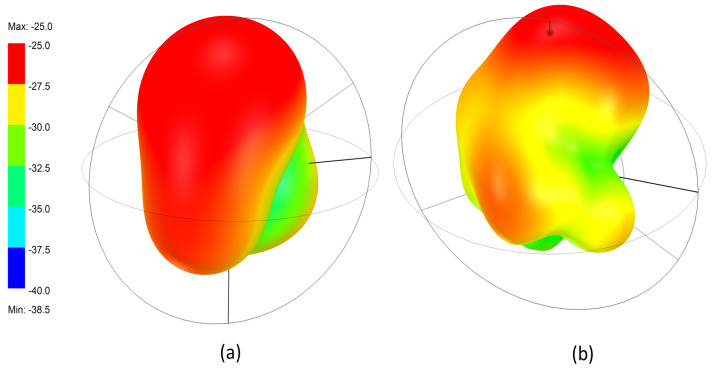
3D radiation patterns of the implantable antenna inside tissue: (**a**) at 1.32 GHz showing a smooth omnidirectional profile, and (**b**) at 2.58 GHz showing a multi-lobed pattern due to higher-order mode effects.

The 3D radiation characteristics of the proposed implantable antenna at both operating frequencies are presented in Figure 15, where Figure 15a corresponds to 1.32 GHz and Figure 15b corresponds to 2.58 GHz. The radiation behavior is strongly influenced by the surrounding high-permittivity and lossy biological tissues, which significantly alter the field distribution compared to free-space antennas.

As shown in Figure 15a, the antenna exhibits a smooth and nearly omnidirectional radiation pattern at 1.32 GHz. The energy is distributed uniformly around the antenna, forming a bulb-shaped pattern. This behavior results from the dominant fundamental mode inside the multilayer tissue environment, where electromagnetic waves experience strong dielectric loading and absorption.

The omnidirectional pattern is beneficial for wireless power transfer since the orientation of the implanted device cannot be precisely controlled inside the body. Regardless of how the antenna rotates or shifts within the tissue, it can still receive power from the external transmitter. The maximum realized gain at this frequency is approximately −25 dB, which aligns with typical values for compact implantable antennas embedded in lossy biological media. Figure 15b illustrates the 3D radiation pattern at 2.58 GHz, where a more complex and multi-lobed distribution is observed. At this higher frequency, the antenna excites higher-order resonant modes, and the electromagnetic waves experience stronger absorption and scattering by the tissue layers. These interactions produce a less uniform pattern with multiple small lobes and slight directivity.

Despite the irregularities, the antenna maintains a clear outward-radiating region, which is advantageous for data telemetry. A more directive nature at 2.58 GHz helps improve communication quality with the external receiver, allowing the antenna to radiate more effectively through tissue in the intended direction.

**Figure 16:**
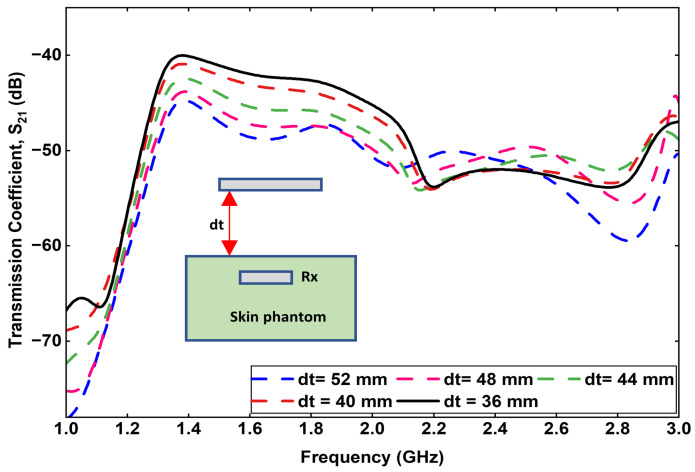
Variation in transmission coefficient by changing the spacing between the Tx and skin phantom (dt).

To further analyze the wireless power transfer characteristics, the spacing (*d_t_*) between the external Tx antenna and the skin phantom was varied, and its impact on the transmission coefficient ($S_{21}$) was examined. The parameter *d_t_* represents the distance separating the Tx antenna from the skin surface, as illustrated in Figure 16.

The variation in $S_{21}$ with different values of *d_t_*, ranging from 36 mm to 52 mm, is presented in Figure 16. It can be observed that when the Tx antenna is positioned at a distance of 36 mm from the skin phantom, the transmission coefficient is approximately −40 dB at 1.32 GHz, indicating strong coupling between the Tx and the implantable Rx antenna. As the spacing *d_t_* increases from 36 mm to 52 mm in 4 mm increments, the $S_{21}$ value gradually decreases to −45 dB. This reduction corresponds to a minimal degradation in power transfer efficiency, which demonstrates that the proposed antenna system maintains stable far-field coupling performance even with moderate variations in Tx placement. The small variation in $S_{21}$ with increasing *d_t_* confirms the robustness of the proposed design for practical biomedical applications, where slight positional deviations between the external power transmitter and the implanted device are unavoidable. The results validate that efficient power transfer can still be achieved under realistic operational conditions.

**Figure 17:**
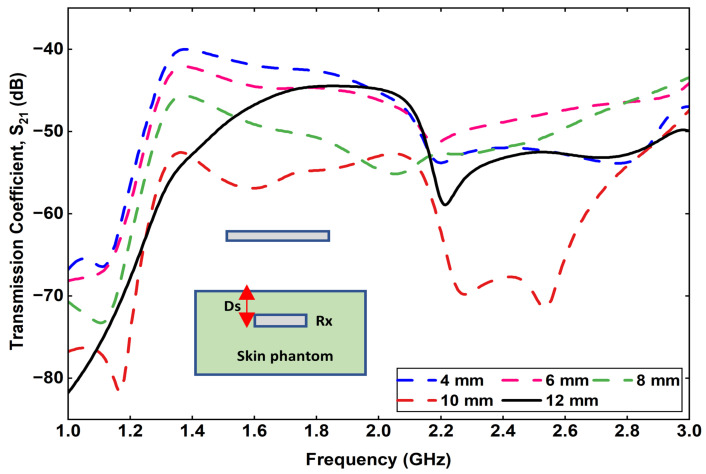
Effect of varying implantation depth (D_s_) on the transmission coefficient.

**Figure 18:**
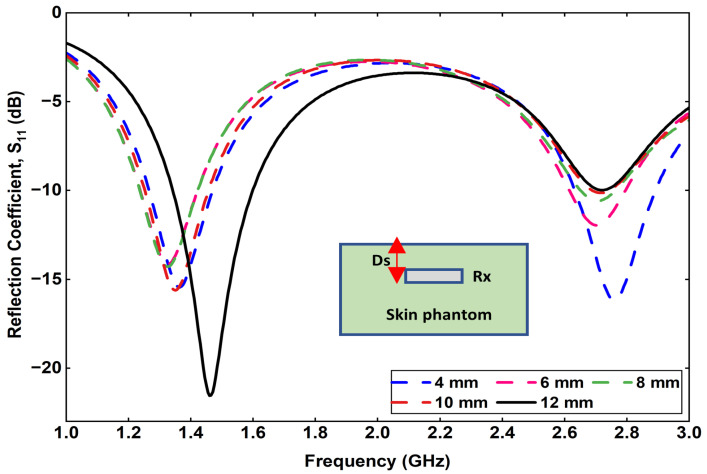
Effect of varying implantation depth (D_s_) on the reflection coefficient.

Another important parameter influencing the wireless link performance is the implantation depth (*D_s_*) of the Rx antenna within the skin phantom. The parameter *D_s_* represents the distance between the skin surface and the implanted Rx antenna, as illustrated in Figure 17. To investigate its impact, the value of *D_s_* was varied from 4 mm to 12 mm in increments of 2 mm, while keeping the Tx–skin spacing constant. The corresponding variation in transmission coefficient ($S_{21}$) is shown in Figure 18. It is observed that as the implantation depth increases, the coupling efficiency decreases significantly. Specifically, when the Rx is positioned at a depth of 12 mm within the skin phantom, the $S_{21}$ value drops to approximately −57 dB at 1.32 GHz, indicating reduced received power due to higher tissue attenuation and dielectric losses.

**Figure 19:**
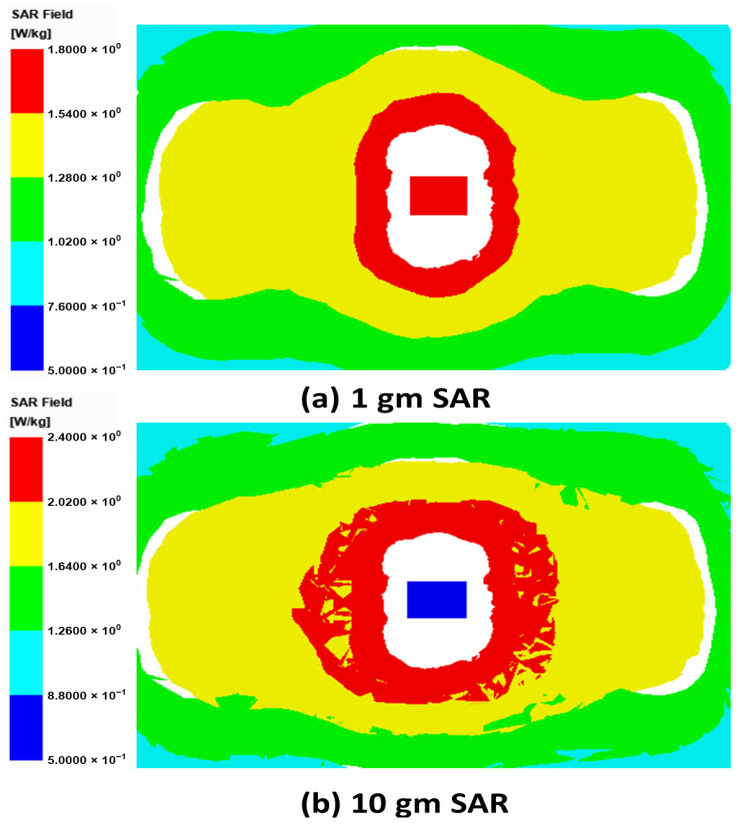
SAR distribution in a multilayer tissue.

The SAR quantifies the rate at which radio-frequency (RF) energy emitted by the implant antenna is absorbed by the surrounding biological tissues. SAR analysis was performed to ensure that the proposed WPT system adheres to the Federal Communications Commission (FCC) and International Commission on Non-Ionizing Radiation Protection (ICNIRP) safety standards [19]. When the transmitter operated at an input power of 1 W, the simulated 1-g averaged SAR reached approximately 10 W/kg, which exceeds the regulatory safety limits. By reducing the transmitter power to 0.1 W, the SAR levels were significantly lowered, bringing them within the acceptable exposure range. As illustrated in Figure 19, the computed 1-g and 10-g averaged SAR values are 1.2 W/kg [Figure 19a] and 2.4 W/kg [Figure 19b], respectively. Both values lie well within the permissible limits of 1.6 W/kg (1 g) and 2.0 W/kg (10 g), confirming the electromagnetic safety of the proposed implantable WPT system.

## 4. Conclusions

In this work, a compact dual-band implantable receiving (Rx) antenna has been designed, analyzed, and optimized for wireless power transfer and data communication in biomedical implant systems. The antenna operates efficiently at two frequency bands centered at 1.32 GHz and 2.58 GHz, corresponding, respectively, to the power transfer and telemetry communication links. The design employs a double-slot configuration on the radiating patch, an additional fine-tuning slot, and a via connection to the ground plane to achieve size reduction and stable dual-band operation. The use of a high-permittivity substrate (Rogers RT/Duroid 6010) with low dielectric loss enables significant miniaturization while maintaining acceptable radiation performance within lossy tissue environments.

The antenna evolution process, carried out in five design steps, demonstrates the influence of each structural modification on impedance matching and resonance tuning. Parametric analyses of the feed position, via location, and via diameter provide a comprehensive understanding of the antenna’s sensitivity to geometric parameters and their effects on the reflection coefficient ($S_{11}$) characteristics. The optimized Rx antenna achieves impedance bandwidths of 1.23–1.42 GHz and 2.47–2.70 GHz, effectively covering the desired operational bands.

The wireless power transfer performance between the external Tx antenna and the implantable Rx antenna is evaluated through the transmission coefficient ($S_{21}$). A power transfer efficiency of −40 dB is achieved at a 40 mm separation distance, with the Rx located in the far-field region of the Tx. The measured realized gains of −25 dB at 1.32 GHz and −25.8 dB at 2.58 GHz are consistent with the expected values for implantable antennas, given the high dielectric loading of human tissues. The SAR analysis confirms that the proposed design complies with IEEE and ICNIRP safety standards, ensuring safe operation within biological environments.

Overall, the proposed dual-band implantable antenna demonstrates reliable performance for far-field wireless power transfer and data communication. Its compact structure, wide impedance bandwidths, and low SAR values make it a strong candidate for next-generation implantable biomedical devices such as biosensors, stimulators, and wireless monitoring systems. Future work will focus on experimental validation in realistic tissue phantoms, optimization for multi-implant communication, and integration with rectifier and telemetry circuitry to realize a complete wireless implantable platform.

## Data Availability

The original contributions presented in this study are included in the article. Further inquiries can be directed to the corresponding author.
